# Biologic Effect of Hydrogen Sulfide and Its Role in Traumatic Brain Injury

**DOI:** 10.1155/2020/7301615

**Published:** 2020-12-22

**Authors:** Jiaxin Zhang, Shaoyi Zhang, Haiyan Shan, Mingyang Zhang

**Affiliations:** ^1^Institute of Forensic Sciences, School of Biology & Basic Medical Sciences, Soochow University, Suzhou, China; ^2^Department of Obstetrics and Gynecology, The Affiliated Suzhou Hospital of Nanjing Medical University, Suzhou, China

## Abstract

Ever since endogenous hydrogen sulfide (H_2_S) was found in mammals in 1989, accumulated evidence has demonstrated that H_2_S functions as a novel neurological gasotransmitter in brain tissues and may play a key role in traumatic brain injury. It has been proved that H_2_S has an antioxidant, anti-inflammatory, and antiapoptosis function in the neuron system and functions as a neuroprotective factor against secondary brain injury. In addition, H_2_S has other biologic effects such as regulating the intracellular concentration of Ca^2+^, facilitating hippocampal long-term potentiation (LTP), and activating ATP-sensitive K channels. Due to the toxic nature of H_2_S when exceeding the physiological dose in the human body, only a small amount of H_2_S-related therapies was applied to clinical treatment. Therefore, it has huge therapeutic potential and has great hope for recovering patients with traumatic brain injury.

## 1. Introduction

Traumatic brain injury (TBI) is the leading cause of high mortality and high morbidity in young adults and a major cause of death and disability across all ages in all countries, with a huge burden on individuals and society in economic and healthy development. Significant progress has been made in providing treatment for TBI and achieving more equitable and sustained improvements across health services. Our group demonstrated for the first time that decreased endogenous hydrogen sulfide (H_2_S) levels and H_2_S synthesis enzyme cystathionine beta-synthase (CBS) expression in the brain were associated with increased lesion volume and mortality after TBI and the protective effect of exogenous H_2_S on TBI [1–3]. Accumulating evidence has demonstrated the protective effect of H_2_S on TBI [1, 2, 4–8].

H_2_S that smells disgusting odor is classified as the third gaseous transmitter following carbon monoxide and nitric oxide. Nowadays, evidence has demonstrated that H_2_S was found to be produced endogenously in various parts of the body such as the heart, blood, and central nervous system (CNS) [9, 10]. Moreover, it also refers to the actions of H_2_S which is involved in the regulation of intracellular signaling molecules, ion channel function, and the release and function of amino acid neurotransmitters [11]. However, it was recently proposed that the neurological actions of H_2_S were continuously modulated primarily by circulating sulfide rather than by endogenous production [12]. This was disproved based on recent studies showing that H_2_S found in the CNS is more likely to be derived directly from the brain than from the blood [13, 14]. Moreover, investigations over the past two decades have shown that the concentration of H_2_S has a detectable change going up or down which is related to brain injury. These results have shown that brain-derived H_2_S seems to serve as an important regulatory mechanism in the growth and development of neurons and the protection of neurons against brain injury.

In TBI, primary damage occurs at the time of impact and the damage is preventable but not treatable. The process will continue to cause following mitochondrial dysfunction, immune responses, the release of excitatory neurotransmitters, cerebrovascular dysfunction, and others that constitute the secondary injury [15]. In order to verify whether H_2_S has the neuroprotective effect after injury, exogenous H_2_S as an endogenous donor has been added into preclinical trials. Most of the neuroprotective drugs tested in mice have failed in human clinical trials because they target a single factor, which mediates secondary injury in TBI. Whether exogenous H_2_S can affect brain-derived H_2_S and has multiple neuroprotective effects on the secondary injury after TBI, it needs more evidence to verify. Therefore, this article focuses on the effective molecular mechanism of H_2_S in TBI and puts forward some views on future research.

## 2. The Severity of TBI

In the clinical setting, the Glasgow Coma Scale (GCS) is a 15-point behavioral observation scale that defines severity based on eye, verbal, and motor response. On the basis of direct observation of a limited number of objective variables, the GCS sums three scores to produce total scores of 3–8 (severe TBI), 9–12 (moderate TBI), and 13–15 (mild TBI). This scoring standard is also not absolute for dividing the severity of TBI. Moreover, there are still some limitations in judging some situations such as patients who are intubated and sedated or paralyzed and a wide range of injury magnitude represented by GCS scores of 3–8 [16]. Patients with GCS scores of 13 were considered moderate TBI for purposes in some studies. Moreover, computed tomography (CT) was used to assess the severity of TBI and a combination of clinical and imaging variables demonstrated a strong correlation with outcome when evaluated using the databases for several clinical trials. Subsequently, Magnetic Resonance Imaging (MRI) was also used as imaging evidence to participate in the assessment of damage. The Centers for Disease Control and Prevention provides one or more of the conditions about changes in consciousness and memory to define the severity of TBI. Therefore, other aforementioned clinical practice guidelines also incorporate structural imaging, duration of loss of consciousness and posttraumatic amnesia, and the GCS in their criteria for classifying injury severity (Table 1).

The establishment of the animal model is an excellent platform to delineate key injury mechanisms that associate with types of injury (concussion, contusion, and penetration injuries) that occur clinically for the investigation of mild, moderate, and severe forms of TBI [17], considering that the evaluation criteria for clinical use cannot be applied to the preclinical models. Therefore, well-defined grading guidelines for defining mild, moderate, and severe TBI in the rodent model are needed. Tissue loss about lesion size analysis is also recognized as a parameter to grade TBI severity. A number of studies utilized loss of cortical and hippocampal tissue to define TBI severity. Mild TBI has been defined as lesions confined to the cortical layer, a moderate injury was associated with considerable cortical tissue loss with little to no overt hippocampal loss, and severe TBI was often defined as extensive overt hippocampal lesions along with cortical tissue loss [18, 19]. There are several common methods for establishing rodent TBI models, including the fluid percussion injury, controlled cortical impact (CCI), and weight-drop model [15]. CCI is well regarded because it allows researchers to quantify the relationship between measurable engineered parameters (e.g., force, velocity, and depth of tissue deformation) and the extent of (either functional and/or tissue) impairment [20]. The CCI model was originally developed to investigate moderate to severe TBI and is infrequently used to mimic mild TBI because of the necessity of a craniectomy. However, modified versions of CCI have now been developed which means that CCI can induce the TBI model with different degrees of injury. According to previous articles, for mild TBI, the depth of impact in the cortex ranged from 0.1 to 1.0 mm and velocity ranged from 3.0 to 6.0 m/s; for moderate TBI, the depth of impact in the cortex ranged from 0.5 to 3.0 mm and velocities of 1.5–6.0 m/s, and severe injury used impact depths of 0.5–2.0 mm and velocities of 3.0–6.0 m/s [15, 20–23]. Many studies use a composite neurological evaluation to assess the severity of motor deficits following TBI, but the problem is still that the assessment criteria cannot be unified. Thus, CCI affords pristine ability to analyze the biomechanical parameters of injury of interest in TBI research.

Although each case of TBI is unique and affected individuals display different degrees of injury (Table 1), we will discuss some of the common underlying neurochemical and molecular mechanisms and common secondary events following different severity of TBI.

## 3. Production and Storage of Endogenous H_2_S

Under physiological conditions, endogenous H_2_S in human bodies is generated via enzymatic and nonenzymatic pathways [24]. H_2_S via nonenzymatic pathways is mainly produced by the decomposition of an inorganic substance, which accounts for a little percentage of H_2_S production. The main generation of H_2_S in the human body mainly depends on the enzymatic pathways using L-cysteine as the substrate. Three types of enzymes play a key role in these pathways, including cystathionine-*β*-synthase (CBS), cystathionine-*γ*-lyase (CSE), and 3-mercaptopyruvate transferase (3-MST) [25]. CBS hydrolyzes cysteine to produce H_2_S, with L-serine as the by-product. Furthermore, CBS could catalyze the condensation of cysteine and homocysteine to form cystathionine and H_2_S. The release of H_2_S could also depend on the reaction on the thiol of cysteine by CBS catalyzing to form s-thiolate. In addition, cysteine also may be hydrolyzed by CSE to produce H_2_S with the concomitant production of pyruvate and ammonia. In addition to CBS and CSE, 3-MST as a pyridoxal-5'-phosphate-independent enzyme is also involved in the production of H_2_S and is identified in the neurons. 3-MST acts together with cysteine aminotransferase (CAT) to generate H_2_S from cysteine (Cys) in the presence of *α*-ketoglutarate. The additional pathway for H_2_S biosynthesis has been reported that 3-MST along with D-amino acid oxidase (DAO) produces H_2_S from D-cysteine by the interaction of mitochondria and peroxisomes which occurs mainly in the cerebellum and the kidney. Both CBS and CSE are pyridoxal-5′-phosphate-dependent enzymes, but they mediate, respectively, the production of H_2_S in different tissues and organs. CBS is expressed mainly in the central nervous system, liver, and kidney while CSE is expressed mainly in the vascular smooth muscle, nonvascular smooth muscle, and a little in the liver, kidney, uterus, placenta, pancreas, and other organs [26]. The location of these enzymes in different tissues is very important because the regulation of endogenous H_2_S production can be achieved by targeting each enzyme separately or simultaneously.

Under physiological conditions (pH = 7.4), H_2_S largely exists in two forms: the neutral molecular form (H_2_S) and an ionic form (HS^−^). The two forms are able to transform into each other and maintain the dynamic equilibrium at a ratio of one to two [27]. Although endogenous H_2_S can be synthesized and released immediately, the storage forms of H_2_S are also known. The acid-labile pool and the sulfane sulfur pool, which include hydrodisulfides/persulfides, are accepted storage forms of H_2_S ([28]), of which the acid-labile pool consists of iron-sulfur-containing proteins located in mitochondrial enzymes and can only release H_2_S at an acid pH of 5.4. The sulfane sulfur pool is localized in the cytoplasm and releases H_2_S under reducing conditions of pH 8.4 [29]. Because mitochondria are not in the acidic condition, acid-labile sulfur may not be a physiologic source of H_2_S. Free H_2_S is immediately absorbed and stored as bound sulfur. 3-MST and CAT are located in the mitochondria and can produce H_2_S from cysteine in the brain [30]. It is speculated that H_2_S produced by the 3-MST/CAT enzymatic pathway is stored in the bound sulfane sulfur pool [9, 31].

## 4. The Change of Endogenous Hydrogen Sulfide in TBI

Up to now, multiple experiments have been designed to examine and describe the change of H_2_S concentration after TBI both in animal models and in human patients. Our group demonstrated for the first time that the concentration of H_2_S presented a dynamic change in the TBI model. The level of endogenous H_2_S and CBS expression in the blood and brain exhibits a downtrend after TBI [2, 3]. Recently, our group also reported that pretreated with NaHS, a H_2_S donor, a limited lesion volume in the ipsilateral cortex has been observed in the TBI model of mice [1]. Jiang et al. reported NaHS treatment increased the H_2_S level of brain tissue and endogenous antioxidant enzymatic activities and decreased oxidative product levels in the brain tissue of TBI-challenged rats [5]. Campolo et al. reported that ATB-346, a hydrogen sulfide-releasing derivative of naproxen, significantly reduced the severity of inflammation and restored neurotrophic factors that characterized the secondary events of TBI [4]. Karimi et al. reported that NaHS has a neuroprotective effect on TBI-induced memory impairment in rats [6]. Xu et al. reported that NaHS restores mitochondrial function and inhibits autophagy by activating the PI3K/Akt/mTOR signaling pathway to improve functional recovery after TBI [7]. The experimental evidence indicates that H_2_S functions as an important neuroprotective mediator in TBI models and may offer clinical therapy for TBI treatment.

## 5. Biologic Effect of Exogenous Hydrogen Sulfide in TBI

**Figure 1:**
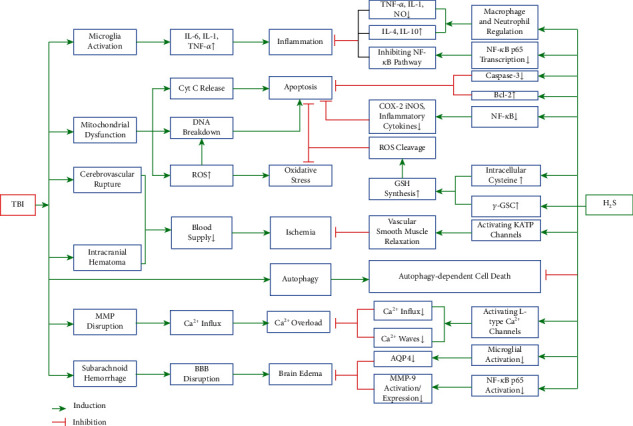
The potential therapeutic effects of H_2_S on TBI. The diagram shows the mechanisms of TBI-induced pathological change in the brain tissues and the nervous system, along with part of the neuroprotective pathways of H_2_S. Note: ↑: upregulated; ↓: downregulated.

Ever since H_2_S was described for the first time in 1713, the toxicity of H_2_S to the human has become the focus of everyone's attention. H_2_S is often considered as one of the most unusual and reliable toxic gas [32]. H_2_S exerts its toxic effects characterized by acute central neurotoxicity, pulmonary edema, conjunctivitis, and odor perception followed by respiratory paralysis which is mainly due to the binding of sulfide to cytochrome c oxidase in mitochondria. Clinical symptoms of acute sulfide poisoning are featured by memory loss and brain dysfunction, which can be explained by the process that excess H_2_S in brain tissue lowers the level of neurotransmitters by inhibiting monoamine oxidase [10]. However, in recent years with the increasing study on H_2_S, its biological effect is gradually being recognized, especially its neuroprotective ability after TBI (Figure 1).

### 5.1. Anti-Inflammatory Role of H_2_S

Neuroinflammation, which is characterized by the activation of glial cells, recruitment of neutrophils and macrophages, and upregulation of cytokines, adhesion factors, and chemokines, can be triggered from the surrounding to the site of injury and cause secondary neuronal injury after TBI [33, 34]. The pioneering work revealed the anti-inflammatory properties of H_2_S in different disease models. In the TBI model, the neuroprotective effect of H_2_S in inflammatory is generally achieved by the regulation of macrophages and neutrophils. H_2_S inhibits the release of proinflammatory factors such as tumor necrosis factor-*α* (TNF-*α*), interleukin-1*β* (IL-1*β*), and NO from astrocytes and microglial cells [10] and increases anti-inflammatory cytokines like interleukin-4 (IL-4) or interleukin-10 (IL-10) [35]. One of the possible anti-inflammatory mechanisms is that H_2_S inhibits the iNOS, NF-*κ*B, ERK, and p38 MAPK signaling pathways [36]. NF-*κ*B transcription factors are ubiquitously expressed in mammalian cells and are known to upregulate the expressions of cytokines and chemokines. The NF-*κ*B family (also known as the Rel family) consists of five members: p50, p52, p65 (also known as Rel A), c-Rel, and Rel B. Inflammation begins to be mediated when activated NF-*κ*B enters the nucleus to induce transcription of a myriad of genes. It has been reported that ATB-346, a hydrogen sulfide-releasing cyclooxygenase inhibitor, significantly reduced the translocation of the p65 submit following TBI in rats [4]. Xiang et al. showed that H_2_S alleviates lipopolysaccharide- (LPS-) induced inflammatory via inhibition of NF-*κ*B p65 translocation [37]. In addition, H_2_S can also polarize microglia to an anti-inflammatory phenotype (M2) by activating calmodulin-dependent protein kinase *β*- (CaMKK*β*-) dependent AMP-activated protein kinase (AMPK) [38]. However, the role of H_2_S in the inflammatory reaction still remains controversial. Some groups have observed that H_2_S plays a proinflammatory role in severe burn injury and LPS-induced inflammation [39, 40]. Consequently, H_2_S is currently accepted to be one of the pivotal factors that regulate the inflammatory reaction following TBI, but the precise mechanism that H_2_S modulates inflammation remains to be elucidated.

### 5.2. Antioxidant Role of H_2_S

During the process of TBI, the physical and secondary damage at the trauma site result in mitochondrial dysfunction and changes in membrane permeability, causing the breakdown of the balance between the generation and elimination of intracellular reactive oxygen species (ROS) (Campolo et al.). Then, tremendous intracellular accumulation of ROS induces cytotoxicity, causing a large amount of cell death.

Oxygen consumption of brain tissue accounts for nearly 20% of the entire body, making brain tissue more sensitive to oxidative stress-related disorders such as TBI, stroke, and Alzheimer's disease [40, 41]. Glutathione (GSH), a tripeptide consisting of cysteine, glutamate, and glycine, is a major antioxidant in the cellular defense against oxidative stress, and H_2_S can promote the production of GSH ([42]; Kimura and [43]). The intracellular concentration of cysteine is much lower than the other two substrates of GSH [44], and cysteine exists in the extracellular space in an oxidized form, cystine [45], so the availability of intracellular cysteine significantly determined the rate of GSH synthesis. Evidence has shown that H_2_S reduces cysteine into cystine and activates the cysteine/glutamate antiporter (xc-) which couples the import of cysteine and export of glutamate [46], consequently increasing the intracellular concentration of cysteine. On the other hand, H_2_S is able to potentiate the activity of *γ*-glutamylcysteine synthase (*γ*-GSC), which functions as a rate-limiting enzyme in the production of GSH (Kimura and [43]). In addition, H_2_S may have a synergistic effect with other antioxidative systems including haem oxygenase (HO), superoxide dismutase (SOD), and especially nuclear factor erythroid-2-related factor2 (Nrf2) [47]. More evidence shows H_2_S can induce the upregulation of Nrf2 and drive it into the nucleus, and it can regulate the level of antioxidant enzymes and enhance the antioxidant reaction which is certified in the diverse damage models [48]. Meanwhile, it is reported that H_2_S can reduce the production of prooxidase, which still represents the inhibition of the occurrence of oxidative stress [49]. It is worth pointing out that H_2_S produced by 3-MST, which is localized in the mitochondria, can directly reduce the generation of ROS and protects cells [50]. Our group reported that H_2_S protects against cell damage induced by scratch injury through modulation of the PI3K/Akt/Nrf2 pathway, suggesting H_2_S may have therapeutic efficacy in TBI [51].

### 5.3. Regulation of Cell Death Signaling Mediated by H_2_S

Neuronal cell death following TBI is an important factor in neurological deficits. TBI can trigger localized neuronal apoptosis within a few hours after the trauma. Increased cytosolic free Ca^2+^ can induce the collapse of the mitochondrial membrane potential and the production of free radicals and lipid peroxidation. This leads to further attack on the mitochondrial membranes, which leads to the release of cytochrome C through the outer mitochondrial membrane. Then, cytochrome C enters the cytoplasm, inducing caspase-dependent apoptosis [52]. Meanwhile, ROS produced from dysfunctional mitochondria can also induce neuronal apoptosis by directly causing the breakdown of DNA. As is discussed above, part of the antiapoptotic effect of H_2_S is achieved through the inhibition of the ROS injury. It has been observed that H_2_S is able to inhibit the H_2_O_2_-activated calcium signaling pathways in mouse hippocampal neurons [53]. In the model of oxygen-glucose deprivation/reoxygenation- (OGD/R-) induced neuronal apoptosis, H_2_S is observed to inhibit a ROS-mediated caspase-3 pathway [54]. Ji et al. found that H_2_S preconditioning considerably reduced TUNEL-positive cells and cleaved caspase-3 in the cerebral ischemia/reperfusion injury model, and the protective effects of H_2_S are possibly achieved by the induction of HSP70 through the PI3K/Akt/Nrf2 pathway [55]. Caspase-3, Bcl-2, and Bax protein function as important mediators in the TBI-induced apoptosis [56]. We found that NaHS pretreatment can reverse the effects on the cleaved caspase-3 increase and Bcl-2 decrease in the injured cortex and hippocampus after TBI [1]. In addition, H_2_S plays another antiapoptotic role via the regulation of the NF-*κ*B signaling pathway, which plays a complex role in integrating signaling between and within neurons and glial. H_2_S reduces the expression of NF-*κ*B followed by the decrease of the expression of iNOS, COX-2, and proinflammatory cytokines. Sen et al. showed that H_2_S sulfhydrate, the p65 subunit of NF-*κ*B at cysteine-38 which augments its ability to bind its coactivator RPS3 and the activator/coactivator complex, then stimulates the transcription of antiapoptotic genes [57].

Autophagy is a physiological process that helps maintain a balance between the manufacture of cellular components and breakdown of damaged organelles and other toxic cellular constituents [58]. Sarkar et al. have shown that autophagic clearance is impaired early after TBI and correlates with neuronal cell death [59]. The Nomenclature Committee of Cell Death defines autophagy-dependent cell death as “a form of regulated cell death that mechanistically depends on the autophagic machinery” and “a distinct mechanism of cell death that occurs independently of apoptosis or necrosis” [60, 61]. Autophagy as an attractive therapeutic target can be developed new therapeutic strategies to achieve better outcomes for patients suffering from TBI [62]. Our group has shown that H_2_S regulated autophagy-dependent cell death after TBI [1, 51, 63]. We also showed that 3-MST was mainly located in living neurons and may be implicated in the autophagy of neurons and involved in the pathophysiology of the brain after TBI [8].

### 5.4. Cerebral Vasodilation Mediated by H_2_S

Research conducted in laboratory animals and humans has investigated the effects of TBI on cerebral blood flow (CBF). Many investigations have revealed that focal or global cerebral ischemia occurs frequently by detection within a temporal range from ultraearly to late stages after TBI. The mechanism may be that the rupture of cerebral vessels, nerve parenchyma, and edema formed by intracranial hematoma may block blood flows, resulting in insufficient blood supply. After TBI, CBF autoregulation, which refers to cerebrovascular constriction or dilation in response to increases or decreases in cerebral perfusion pressure (CPP), is impaired or abolished in most patients. The defect of automatic adjustment of cerebral blood flow occurs in an uncertain time after injury, which can occur immediately after the trauma or may develop at other times after injury. Furthermore, about more than one-third of TBI patients show cerebral vasospasm, and in all patients with vasospasm, 50% have hypoperfusion. In this process, the destruction of neuronal membrane and dysfunction of various ion channels located on the membrane can cause disrupted ion homeostasis, during which Na^+^ and Ca^2+^ accumulate in the cytoplasm and a large amount of K^+^ is pumped out. As a result, the brain cells become sensitive to ischemia [64]. Multiple studies have proved that H_2_S dilates cerebral vessels following TBI via activation of the ATP-sensitive K channels (K_ATP_ channels) [5]. According to reports, specific molecular targets of H_2_S appear to be cysteine 6 and 26 in the extracellular portion of the rvSUR1 subunit of the K_ATP_ channel complex. The opening of the K_ATP_ channels inactivates cell membrane polarization and voltage-gated calcium channels, which in turn lead to a reduction in Ca^2+^, which ultimately leads to the relaxation and expansion of blood vessels. In a vitro model of rat aortic tissues, H_2_S activates K_ATP_ channels by inducing the efflux of intracellular potassium and the hyperpolarization of the membrane of vascular smooth muscle cells [65]. Later, another group reported that H_2_S has a similar mechanism of vasodilation in the cerebral cortical pial arteriole of newborn pigs [66]. However, conflicting reports have emerged showing that the contribution of the K_ATP_ channels to H_2_S-induced vasodilation is minimal and that vasodilation is due to metabolic inhibition such as the decrease in ATP, intracellular pH changes, and modulation of Cl^−^/HCO_3_^−^ channels. There are also indications that H_2_S may stimulate vasodilation by liberating ^·^NO from S-nitrosothiols. Although the mechanism of cerebral vasodilation is still unclear, it is certain that hydrogen sulfide has the effect of dilating cerebral blood vessels after TBI.

### 5.5. H_2_S Mediates Intracellular Calcium Concentration

After the primary injury of TBI, the disruption of mitochondrial membrane potential (MMP) and intracellular excessive ROS lead to intracellular calcium accumulation, causing calcium overload-induced neurotoxicity [67]. Ca^2+^ activates lipid peroxidases, proteases, and phospholipases which in turn increase the intracellular concentration of free fatty acids and free radicals. Calcium overload, which is at the core of the cellular and molecular network of secondary neuronal injury, is involved in three neurotoxic cascade reactions leading to neuronal apoptosis or necrosis [64]. It has been noted that H_2_S is capable of regulating Ca^2+^ in all important brain cell types, namely, neurons, microglia, and astrocytes. The physiological concentrations of H_2_S selectively were initially found to be a neuromodulator and facilitate the induction of hippocampal long-term potentiation (LTP) by enhancing the activity of N-methyl-D-aspartate (NMDA) receptors in neurons and increase the influx of Ca^2+^ into astrocytes. H_2_S could raise cytosolic calcium in neurons through the activation of L-type Ca^2+^ channels. Some groups have observed a concentration-dependent H_2_S-induced Ca^2+^ elevation in neurons, and such effects of H_2_S on cytosolic Ca^2+^ concentration can be attenuated by the antagonist of L-type calcium channel and N-methyl-d-aspartate receptor [68, 69]. In TBI, H_2_S released from neurons or glia in response to neuronal excitation activates Ca^2+^ channels of astrocytes, inducing Ca^2+^ waves that propagate to the neighboring astrocytes [70], and H_2_S may mediate signals between neurons and glia. However, there are still some reports suggesting that H_2_S elevates neuronal Ca^2+^ concentration and may exacerbate the formation of calcium overload in secondary neuronal injury [64].

### 5.6. H_2_S Attenuates the TBI-Induced Brain Edema

Brain edema, which is usually formed in the early brain injury following subarachnoid hemorrhage, can result in brain swelling and increased intracranial pressure followed with neuronal cell death, herniation, and death [71]. Our group reported that TBI significantly increases the percentage of water content in the injured ipsilateral cortex, leading to the formation of TBI-induced brain edema [1]. It is also reported that exogenous H_2_S treatment can effectively ameliorate the development of TBI-induced brain edema [1]. Another group demonstrated that the effect of H_2_S on the inhibition of brain edema formation might be involved with alleviating blood-brain barrier (BBB) disruption and reducing aquaporin-4 (AQP4) expression, both of which are induced by matrix metalloprotease- (MMP-) mediated tight junction proteins (TJPs) [72]. Classically, brain edema was thought to be triggered by the disruption of BBB integrity in the acute stage of subarachnoid hemorrhage [73]. Then, the degeneration of the basal lamina and the increased permeability of BBB result in the extracellular accumulation of water in the cortex [74]. In recent years, however, multiple investigations demonstrated that the dysfunction of ion and water channels plays a vital role in glial and neuron swelling [75]. Evidence showed that the deletion of AQP4, a main water channel protein concentrated on the astrocyte endfeet, protects BBB integrity by reducing inflammatory responses due to the upregulation of PPAR-*γ* expression and attenuation of proinflammatory cytokine release [76]. In addition, studies also proved that H_2_S attenuates brain edema by inhibiting the expression/activation of MMP-9, which is possibly associated with the regulation of NF-*κ*B p65 activation [72]. It is now demonstrated that H_2_S may attenuate the AQP4-induced cellular edema via inhibition of microglial activation and suppression of proinflammatory cytokine release in the cortex, but the underlying mechanisms of H_2_S alleviating brain edema through cellular pathways still demand further investigation.

### 5.7. Other Mechanisms of H_2_S following TBI

Apart from the biologic effects of H_2_S following TBI mentioned above, the underlying mechanisms of H_2_S are reflected in many other aspects. It was found that H_2_S facilitates hippocampus long-term potentiation via mediation of the N-methyl-D-aspartate receptor in the H_2_S-activated cAMP/PKA pathway [11, 77]. Moreover, H_2_S also counteracts glutamate-mediated excitotoxicity in the secondary injury following TBI via activation of K_ATP_ channels, which may directly result in neuronal glutamate release in response to calcium influx after TBI [64].

According to all that have been mentioned above, the mechanisms of H_2_S following TBI can be summarized in Table 2.

## 6. Conclusion

Although H_2_S has multiple biologic effects as a neuroprotective gasotransmitter and considerable part of them has already been confirmed through experiments in laboratory, applying H_2_S-related therapy to clinical treatment still has a long way to go. H_2_S can function as a neuroprotective mediator when it is controlled within physiological dose, while excess H_2_S has chemotoxic and cytotoxic effects on human bodies [79]. H_2_S still remains many potential mechanisms which have not been totally figured out, but it is beyond doubt that H_2_S therapy, especially on TBI patients, will become an available treatment option in the near future.

## Figures and Tables

**Table 1:** Characteristics of different degrees of TBI in different species.

Species	
Human	*Mild TBI* (1) Any period of loss of consciousness up to 30 min (2) Posttraumatic amnesia not exceeding 24 h (3) Any period of confusion or disorientation (4) Transient neurological abnormalities (5) A GCS score of 13–15 (6) Normal structural imaging (7) Postconcussion symptoms may resolve during 12 weeks
	*Moderate TBI* (1) A possible loss of consciousness lasting up to a few hours (2) Confusion lasting from days to weeks (3) Physical, cognitive, and/or behavioral impairments lasting for months (1) Abnormal structural imaging (2) A GCS score of 9–12
	*Severe TBI* (1) Sustained loss of consciousness (>24 h) (2) Surviving patients exhibiting chronic physical and emotional disabilities (3) Abnormal structural imaging (4) A GCS score of less than 9

Mouse	*Mild TBI* (1) CCI: depth: 0.1–1.0 mm; velocity: 3.0–6.0 m/s (2) Tissue loss: lesions confined to the cortical layer (3) Cortical depression <0.5 mm, velocities <4.0 m/s
	*Moderate TBI* (1) CCI: depth: 0.5–3.0 mm; velocity: 1.5–6.0 m/s (2) Tissue loss: considerable cortical tissue loss with little to no overt hippocampal loss (3) Cortical depression 1.0–1.5 mm, velocities 4.0–5.0 m/s
	*Severe TBI* (1) CCI: depth: 0.5–2.0 mm; velocity: 3.0–6.0 m/s (2) Tissue loss: extensive overt hippocampal lesions along with cortical tissue loss (3) Cortical depression >2.0 mm, velocities >5.0 m/s

Note: TBI: traumatic brain injury; CCI: controlled cortical impact; GCS: the Glasgow Coma Scale.

**Table 2:** Summary of the biologic effects of hydrogen sulfide after TBI.

Effects	Mechanisms	References
Anti-inflammation	Inhibiting the TNF-*α*, IL-1*β*, and NO	[10]
	Increasing the IL-6 and IL-10	[35]
	Inhibiting the iNOS, NF-*κ*B, ERK, and p38 MAPK pathways	[36]

Antioxidation	Activation of cysteine/glutamate antiporter	[46]
	Activation of *γ*-GCS	(Kimura and [43])
	Cooperating with HO, SOD, and Nrf2 antioxidative system	(Kimura and [43])
	Decreasing the production of the prooxidase	(Kimura and [43])

Antiapoptosis	Inhibiting the H_2_O_2_-activated calcium pathways	[53]
	Reducing caspase-3 and increasing Bcl-2	[54]
	Regulating the NF-*κ*B signaling pathway	[57]

Regulating autophagy-dependent cell death	Reducing Beclin-1 and LC-3 LC3-positive cells were partly colocalized with PI	[1] [8] [63] [51]

Vasodilation	Activating the K_ATP_ channels (CSE-generated H_2_S) Liberating NO from S-nitrosothiols	[65] [78]

Ca^2+^ modulation	Activating the L-type Ca^2+^ channels	[68]
	Inducing Ca^2+^ waves	[70]

Attenuating edema	Alleviating BBB disruption and reducing AQP4 expression	[72]

Facilitating LTP	Potentiating the NMDA receptor	[77]

Antiexcitotoxicity	Reducing glutamate release after TBI	[64]

## Abbreviations

3-MST: 3-Mercaptopyruvate transferase
*γ*-GCS: *γ*-Glutamylcysteine synthase
AMPK: AMP-activated protein kinase
AQP4: Aquaporin-4
ATB-346: 2-(6-Methoxynapthalen-2-yl)-propionic acid 4-thiocarbamoyl-phenyl ester
ATP: Adenosine triphosphate
BBB: Blood-brain barrier
Bcl-2: B-cell lymphoma-2
CaMKK*β*: Calmodulin-dependent protein kinase *β*
cAMP: Cyclic adenosine monophosphate
CAT: Cysteine aminotransferase
CBF: Cerebral blood flow
CBS: Cystathionine-*β*-synthase
CCI: Controlled cortical impact
CNS: Central nervous system
CPP: Cerebral perfusion pressure
CSE: Cystathionine-*γ*-lyase
CT: Computed tomography
Cys: Cysteine
DAO: D-Amino acid oxidase
ERK: Extracellular signal-regulated kinase
GCS: Glasgow Coma Scale
GSH: Glutathione
H_2_O_2_: Hydrogen peroxide
H_2_S: Hydrogen sulfide
HO: Haem oxygenase
HSP70: Heat shock protein 70
IL-1*β*: Interleukin-1*β*
IL-4: Interleukin-4
IL-10: Interleukin-10
INOS: Inducible nitric oxide synthase
LPS: Lipopolysaccharide
LTP: Long-term potentiation
MAPK: Mitogen-activated protein kinase
MMP: Mitochondrial membrane potential
MMPs: Matrix metalloproteases
MRI: Magnetic Resonance Imaging
mTOR: Mammalian/mechanistic target of rapamycin
NaHS: Sodium hydrosulfide hydrate
NF-*κ*B: Nuclear factor-kappa B
NMDA: N-Methyl-D-aspartate
NO: Nitric oxide
Nrf2: Nuclear factor erythroid-2 related factor2
OGD/R: Oxygen-glucose deprivation/reoxygenation
PI: Propidium iodide
PI3K: Phosphoinositide 3-kinase
PKA: Protein kinase A
PPAR-*γ*: Peroxisome proliferator-activated receptors *γ*
ROS: Reactive oxygen species
SOD: Superoxide dismutase
TBI: Traumatic brain injury
TJPs: Tight junction proteins
TNF-*α*: Tumor necrosis factor-*α*
TUNEL: Terminal deoxynucleotidyl transferase-mediated dUTP-biotin in situ nick-end labeling.
